# An organizing feature of bumble bee life history: worker emergence promotes queen reproduction and survival in young nests

**DOI:** 10.1093/conphys/coab047

**Published:** 2021-06-29

**Authors:** Erica Sarro, Penglin Sun, Kerry Mauck, Damaris Rodriguez-Arellano, Naoki Yamanaka, S Hollis Woodard

**Affiliations:** Department of Entomology, The University of California Riverside, 900 University Ave., Riverside, CA 92521, USA

**Keywords:** Bumble bee, juvenile hormone, nesting success, queen, reproduction, social insect

## Abstract

Bumble bee queens initiate nests solitarily and transition to living socially once they successfully rear their first cohort of offspring. Bumble bees are disproportionately important for early season pollination, and many populations are experiencing dramatic declines. In this system, the onset of the social stage is critical for nest survival, yet the mechanisms that facilitate this transition remain understudied. Further, the majority of conservation efforts target the social stage of the bumble bee life cycle and do not address the solitary founding stage. We experimentally manipulated the timing of worker emergence in young nests of bumble bee (*Bombus impatiens*) queens to determine whether and how queen fecundity and survival are impacted by the emergence of workers in the nest. We found that queens with workers added to the nest exhibit increased ovary activation, accelerated egg laying, elevated juvenile hormone (JH) titres and also lower mortality relative to solitary queens. We also show that JH is more strongly impacted by the social environment than associated with queen reproductive state, suggesting that this key regulator of insect reproduction has expanded its function in bumble bees to also influence social organization. We further demonstrate that these effects are independent of queen social history, suggesting that this underlying mechanism promoting queen fecundity is reversible and short lived. Synchronization between queen reproductive status and emergence of workers in the nest may ultimately increase the likelihood of early nesting success in social systems with solitary nest founding. Given that bumble bee workers regulate queen physiology as we have demonstrated, the timing of early worker emergence in the nest likely impacts queen fitness, colony developmental trajectories and ultimately nesting success. Collectively, our findings underline the importance of conservation interventions for bumble bees that support the early nesting period and facilitate the production and maintenance of workers in young nests.

## Teaser

The emergence of workers in young bumble bee nests promotes queen physiological changes related to early nesting success. Our findings underline the importance of conservation interventions for bumble bees that support the early nesting period and facilitate the production and maintenance of workers in young nests.

## Introduction

The ability to synchronize life history transitions with changes in the environment is essential to organismal survival and fitness. Disconnects between transient environmental characteristics and behavioural and physiological states can result in fitness declines (Stenseth and Mysterud, 2002). Conversely, closely coordinating key life history shifts with environmental changes allows organisms to better track resource availability and optimize fitness under current ecological conditions. For example, many animals emerge from winter diapause at the onset of spring, when food resources become available, thus aligning their heightened metabolic activity with access to adequate nutrition (Tauber and Tauber, 1976; Koštál, 2006). Behavioural and physiological changes associated with life history transitions are often responses to a suite of internal factors, such as nutritional or circadian state, as well as external cues, such as daylength, temperature and chemical and visual stimuli (Flatt and Heyland, 2011). Often, these external cues are reliable indicators that directly reflect changes in ecological conditions. Identifying the proximate mechanisms that organize life history transitions and understanding the adaptation of these physiological transitions to environmental variation are major goals in conservation physiology research (Sinervo and Svensson, 1998).

In social animals, society members also regulate the behaviour and physiology of one another in ways that promote cohesiveness between group members and support the survival of the group. The most extreme examples of this are seen in eusocial systems, which are defined by their reproductive division of labor, overlapping generations and cooperative brood care (Batra, 1966; Michener, 1969). For example, reproductive division of labour is maintained by signalling among nestmates, such that reproductively dominant females use pheromones and/or aggression to inhibit worker reproduction and reinforce a reproductive skew between queens and workers (Van Oystaeyen *et al.*, 2014). This queen effect on worker reproduction promotes nesting success by reducing intra-nest conflict (Kocher and Grozinger, 2011). This form of social influence, whereby a queen regulates the physiology of her offspring to her benefit (Linksvayer and Wade, 2005), has been studied extensively in eusocial insects (Van Oystaeyen *et al.*, 2014).

Although much is known about how eusocial queens influence worker behaviour and physiology (Keller and Nonacs, 1993; Grüter and Keller, 2016), little work has investigated the ways in which workers influence queens. Bumble bees (genus *Bombus,* family Apidae) are one of several social insect lineages in which queens live and reproduce under both solitary and social conditions. In these systems, nests are first initiated solitarily by queens, then transition to sociality when the first offspring eclose. This form of sociality is also seen in lineages such as some ponerine ants, halictine and xylocopine bees and vespid wasps (Wilson, 1971). In these systems, emergence of the first workers in the nest exposes queens to an array of new social signals not present during the solitary nest-founding stage. In bumble bees, like most other solitary nest-founding social lineages, worker emergence coincides with a transition in queens from performing a broad task repertoire (e.g. brood feeding, foraging, nest maintenance, defence) to almost exclusively producing and laying eggs (Shpigler *et al.*, 2013; Woodard *et al.*, 2013). This transition is likely directly facilitated by the onset of the social environment, as bumble bee queens that are experimentally manipulated to become social through the addition of workers (Sladen 1912; Röseler, 1968; Kwon *et al.*, 2006; Woodard *et al.*, 2013), brood (Kwon and Saeed, 2003; Kwon *et al.*, 2006), conspecific queens (Strange, 2010) or even honey bee workers (Strange, 2010), lay eggs earlier and in greater numbers than solitary queens. Thus, it appears that queen bumble bees synchronize their transition to a more reproductive state with the emergence of helpers in the nest who will rear those offspring. However, the physiological underpinnings of this reproductive acceleration remain unknown. Further, it is unclear what factors influence the onset and persistence of queen reproduction in this pollinator lineage, which might translate to similarly solitary-founding social species.

Bumble bees are one insect lineage for which there is strong evidence of decline (Williams, 1982; Cameron and Sadd, 2020; Colla *et al.*, 2012). Insights into the factors that ensure nesting success are particularly important to derive for this annually nesting lineage. An estimated 25% of species in this group are considered threatened (IUCN Red List). The solitary nest founding stage represents a unique challenge for the social insects in which this occurs, such as bumble bees, because queens are not yet buffered by the social environment and must do all work for the nest, including risking exposure when foraging for resources (Oster and Wilson, 1978). Existing studies suggest that nests at this stage are particularly sensitive to pesticides (Baron *et al.*, 2017; Leza *et al.*, 2018), parasites (Rutrecht and Brown, 2008; Elliott, 2009) and other stressors (Watrous *et al.*, 2019; Mola *et al.*, 2021). Studies on the physiological basis of bumble bee decline are important for understanding the mechanistic drivers of population health and population declines, which can be leveraged to target effective conservation strategies (Woodard, 2017). Examining the physiological impact of the social environment on early nesting queens, specifically, will elucidate the proximate mechanisms shaping population dynamics at this fundamental life stage.

Here, we explored the hypothesis that the emergence of workers in the nest promotes queen physiological changes related to early nesting success, by examining worker regulation of queen survival and reproductive behaviour and physiology in recently-founded nests of the bumble bee *Bombus impatiens*. In the bumble bees, workers do not feed or groom the queen, as is the case in some other social lineages (Naumann, 1991). This provides a unique opportunity to investigate both direct and indirect impacts of workers in the nest, independent of nutritional or hygienic factors. We experimentally manipulated the timing of worker emergence in the nest and measured queen physiological responses to this social manipulation over multiple time scales during the early nesting stage. To assess queen reproductive physiology, we quantified egg laying, degree of ovarian activation and juvenile hormone (JH) titres in hemolymph. JH is a key regulator of ovary development and reproduction in female insects (Wigglesworth, 1934; Roy *et al.*, 2018). We predicted that worker presence would elevate JH titres and expedite ovarian development in social relative to solitary queens, thus facilitating the previously observed acceleration of functional reproduction in bumble bee queens (Röseler, 1968; Kwon *et al.*, 2006; Woodard *et al.*, 2013). We further predicted that JH titres and ovarian activation would be positively associated with one another, irrespective of queen social status, which would indicate that JH has maintained its gonadotropic qualities in bumble bees.

We also experimentally removed brood and/or workers from a separate subset of queens to explore the impact of social history on queen reproduction. Here, we examined the rate of nest re-initiation after a simulated loss of brood and/or workers. In this experiment, we asked whether queens who were historically social would maintain elevated reproductive output relative to previously solitary queens. This was predicated on the idea that social environments might have enduring positive effects on reproduction. If our results support this, it would suggest that queens who successfully rear one set of offspring have a reproductive advantage over those who have not, even if they subsequently lose those offspring and must reinitiate a new nest. Alternatively, the effects of the social environment may be more transitory, in which case a queen who loses her first brood would have no measurable reproductive advantage. To our knowledge, this is the first study to investigate the proximate mechanisms underlying worker-induced queen reproduction in bumble bees and to explore the persistence of social effects on fecundity in an imperilled insect group.

## Materials and methods

### Experiment 1: Queen reproductive physiology in response to the presence of workers

#### Bee rearing and experimental design

We first explored the impact of worker presence on queen reproductive maturation and physiology across time. Thirteen mature *B. impatiens* Cresson colonies (containing a queen and ≥50 workers) were acquired from Koppert Biological Systems (Howell, MI, USA) and kept in the University of California Riverside’s Insectary and Quarantine Facility under dim red light at 27°C and 60% RH. A subset of these colonies were at the developmental stage where new reproductives (queens and males) are produced**.** These colonies were used to source queens for this experiment. The remaining colonies were younger and were used to source female workers for this experiment. Bees were fed *ad libitum* artificial nectar (recipe in Boyle *et al*. 2018) and pollen balls consisting of honey bee-collected, mixed-source pollen (Brushy Mountain Bee Farm, Moravian Falls, NC, USA) blended with artificial nectar.

**Figure 1:**
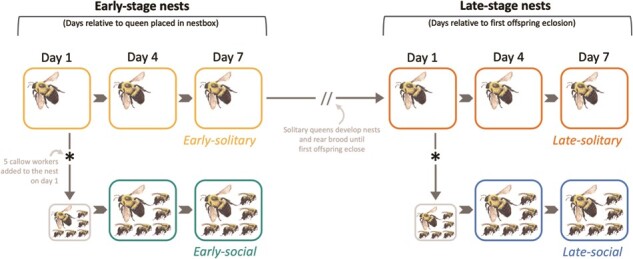
Experimental design implemented in Experiment 1. Early-stage nests were collected (i.e. sacrificed and processed) 1, 4 or 7 days after they were placed in a new next box following their second CO_2_ treatment. Late-stage nests were collected (i.e. sacrificed and processed) 1, 4 or 7 days after their first offspring eclosed in the nest. All males were removed from nests as soon as they were observed. Solitary queens did not receive workers and were solitary for the duration of the experiment. For social queens, 5 callow workers were added to nests either 1 day after the second CO_2_ treatment (early-social) or 1 day after the first male offspring eclosed in the nest (late-social). Early-solitary and late-social groups represent the natural development of sociality in young bumble bee nests, whereas early-social and late-solitary queens represent social manipulations. Colours indicate treatment groups; small boxes indicate days in which workers were added to nests, but no bees or data were collected.

Callow queens (<24 hours old, identified by their silvery appearance) were removed from their natal colonies and arranged in groups containing either a single solitary queen or a queen and five workers, in the following four configurations: early-social (workers added prematurely, before the queen has become reproductive), early-solitary (no workers added at this early stage, before the queen has become reproductive), late-social (workers added after the queen has established a nest and the first adult offspring have eclosed) and late-solitary (no workers added at this later stage, after the queen has established a nest and the first adult offspring have eclosed) (Fig. 1). Throughout the experiment, the cages were kept under dim red lights (which are not visible to bees) at 27°C and 70% relative humidity (RH). All cages were fed the diet described above, with the exception that the first pollen ball provided was coated in honey bee wax.

As in previous studies (Röseler and Röseler, 1988; Amsalem and Grozinger, 2017; Leza *et al.*, 2018), queens were not mated to minimize variation introduced by this process (Baer and Schmid-Hempel, 2005) and to control the number and source of workers in the nests of late-stage queens. All queens were treated with CO_2_ gas for 30 minutes per day at adult ages 12 and 13 days to cause them to bypass diapause and initiate egg laying (Röseler, 1985). CO_2_ treatment is a widely-used technique that causes queens to bypass diapause in a way that is largely indistinguishable from true diapause (Amsalem *et al.*, 2015; Amsalem and Grozinger, 2017). CO_2_-treated queens become reproductive irrespective of mating status (Amsalem *et al.*, 2017; Leza *et al.*, 2018), but produce only haploid male offspring when unmated.

Within 24 hours of the second CO_2_ treatment, 5 callow workers were added to the nests of queens in the early-social group. The remaining queens (early-solitary, late-solitary, late-social) were reared solitarily at this stage. Early-stage nests (i.e. early-social and early-solitary) were collected either 1, 4 or 7 days after CO_2_ treatment (*n* = 6–9). On each nest’s collection day, the queen was sacrificed, hemolymph was collected from the queen (see methods below) and the queen and nest were stored at −80°C. These early-stage time points capture data prior to queen reproductive maturation (early Day 1), at the approximate day social queens begin laying eggs based on preliminary observations (early Day 7) and at an intermediate stage between these two time points to capture the onset of reproductive development (early Day 4). The remaining queens (in the two late-stage groups) were allowed to continue developing their nests and rear their first brood cohort to adulthood (Fig. 1). For the late-social queens, 5 callow workers were added to nests within 24 hours of the first male eclosing in the nest (simulating natural emergence of workers in the nest) following the above methods for worker additions. The remaining queens (late-solitary) were left solitary. Late-stage nests were collected 1, 4 or 7 days after the first male eclosed (*n* = 5–9) to match the length of time queens were exposed to workers in the early-stage treatment groups. In this way, we could directly compare worker effects on queen reproduction in early- versus late-stage queens. Nests were inspected every 1–2 days, and all eclosed males were removed from late-stage nests as soon as they were detected to control the number of adult offspring in nests. Additional sampling details are provided in the Supporting Information.

#### JH-III quantification

Live queens were briefly restrained in plastic marking tubes (Betterbee, Greenwich, CT, USA). Using forceps, heads were swiftly removed to expose the open neck cavity. Using a graduated glass capillary tube, a measured quantity of hemolymph (5–20 ul per bee) was collected from the cavity and placed into a mix of 50 ul acetonitrile (Fisher Scientific A998–4, Waltham, MA, USA) and 50 ul 0.9% sodium chloride solution (Fisher Scientific S271–500) contained within a 9-mm autosampler insert (Fisher Scientific C4010-630) inside an autosampler vial (Fisher Scientific C5000-1W) with a vial cap (Fisher Scientific C5000-54B). This method prevents hemolymph melanization and preserves JH in suspension, following Kai *et al.* (2018). Samples were vortexed and JH was twice extracted into 100-μl volumes of hexanes (Fisher Scientific H306–1) containing 10 ng of citronellol (Sigma-Aldrich W230915, St. Louis, MO, USA) as an internal standard. After each extraction, the JH–hexane–citronellol phase (upper layer) was transferred into a new autosampler vial (with insert and cap). JH extracts were stored at −80°C until they were run on a gas chromatography–mass spectrometry machine according to methods in Kai *et al.* (2018) (see Supporting Information for details). Bees were stored at −80°C until they were processed for dissection.

#### Ovary dissections and measurements

Queen abdomens were soaked in RNAlater®-ICE (Ambion Life Technologies, Austin, TX, USA) at −20°C for 24 hours prior to dissection to enable wet dissection while maintaining fat body RNA integrity for potential future use. RNAlater®-ICE does not cause histological or morphological changes to tissues (Florell *et al.*, 2001). Ovaries were removed and lengths of all eight terminal oocytes were measured with an ocular micrometre. Any oocyte resorption (characterized by yellow colouration and misshapen oocytes lacking a trophocyte, Fig. S1) in terminal oocytes was recorded. Oocyte resorption, in which females reabsorb the nutrients from egg cells that they do not or cannot oviposit, is commonplace in insects, including bumble bees (Bell and Bohm, 1975; Duchateau and Velthuis, 1989). Individuals may resorb eggs due to the lack of suitable oviposition sites, unfavourable environmental conditions or pheromone- or aggression-induced functional sterility (Medler, 1962; Duchateau and Velthuis, 1989). To prevent measurement bias, dissectors were blind to queen treatment group. We also confirmed that workers were non-reproductive when collected by categorically staging all worker ovarioles according to Duchateau and Velthuis (1989). No workers had ovarioles developed beyond stage two, indicating that their ovaries did not contain mature eggs.

#### Body size measurements and nest dissections

The length of bumble bee marginal wing cells is highly correlated to overall body size (Medler, 1962) and was used here as a proxy for body size to be included in statistical analyses. Queen forewings were removed and the marginal cell length of each wing was measured with an ocular micrometre. Cell lengths were averaged together to establish a single measurement per individual. Nests were dissected on dry ice and the number of eggs was recorded.

#### Statistical analyses

All statistical analyses were carried out in R version 4.0.0. Results were visualized using the ggplot2 package (v. 3.3.0; Wickham, 2016). Generalized linear mixed models (GLMMs) were used to determine predictors for JH titre, oocyte length and egg laying rate. GLMMs were carried out using the lme4 package (v. 1.1–23; Bates *et al.*, 2015). Details on model formation are provided in the Supporting Information. For each analysis, Akaike’s Information Criterion for small sample sizes (AICc) was used to select the best-fit model based on the model.sel() function from the car package (v. 3.0-7; Fox and Weisberg, 2019), and the model with the lowest AICc score that was not rank deficient was used for subsequent analyses. *P*-values were acquired using the tab_model() function from the sjPlot package (v. 2.8.3; Lüdecke *et al.*, 2021) and pairwise comparisons were carried out using the lsmeans() function from the lsmeans package (v. 2.30-0; Lenth, 2016) with a Tukey *P*-value adjustment, Welch’s two-sample t-tests using the t.test() function with a Bonferroni *P*-value adjustment or Wilcoxon rank sum exact test using the wilcox.test() function with a Bonferroni *P*-value adjustment. Levene’s test for homogeneity of variance was used to measure variance of samples using the leveneTest() function from the car package.

### Experiment 2: Uncoupling current social environment from social history

#### Bee rearing and experimental design

**Figure 2:**
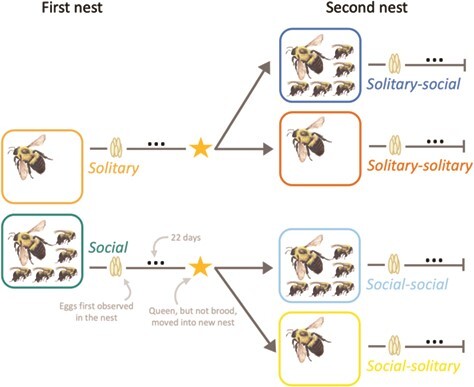
Experimental design implemented in Experiment 2. All queens in Experiment 2 were given the opportunity to initiate 2 independent nests. First nest refers to the nest immediately after the second CO_2_ treatment, pre-brood removal. Second nest refers to the nest immediately following brood removal. For social queens, five callow workers were added to nests either immediately following the queen’s second CO_2_ treatment (first nest) or at the time of transfer to the second nest (second nest). The solitary-social group represents the natural development of sociality in young bumble bee nests, whereas the solitary-solitary, social-solitary and social-social groups represent social manipulations. Colours indicate treatment groups.

We performed a second experiment to examine whether there are persistent effects of the social environment on queen reproductive physiology. Here, bees were sourced from 14 mature *B. impatiens* colonies (also from Koppert Biological Systems) reared as described above, with the exception that nests were kept at ambient room temperature and humidity (22 +/− 2°C; 35 +/− 10% RH). All queens (*n* = 39) were allowed to initiate two consecutive nests to enable repeated measurements. First, queens were randomly assigned to one of four groups: solitary-solitary (queens remained solitary for the duration of the experiment), solitary-social (5 callow workers added to the second nest), social-solitary (5 callow workers added to the first nest, but not transferred to the second nest) and social-social (5 callow workers added to the first nest and subsequently transferred to the second nest) (Fig. 2; *n* = 9–10).

Five callow workers were added to the nests of social-solitary and social-social queens on the same day the queen was added to the first nesting box (adult age 13 days, immediately following second CO_2_ treatment). Nests were monitored every 2–3 days to record the presence or absence of brood. Twenty-two days after the first eggs were observed in the first nest (approximately ¾ of pre-adult worker development time; Cnaani *et al.* 2002), queens, but not brood, were transferred to new nesting boxes. This simulated the loss of brood and removed any related chemical cues from nests. Hereafter, we refer to these pre- and post-brood removal nests as first and second nests, respectively. When queens were transferred to second nests, workers in social-social nests were also transferred to second nests, five callow workers were added to solitary-social second nests, and workers from social-solitary nests were removed and sacrificed. Second nests were monitored until queens re-initiated egg laying, and entire nests were subsequently collected 22 days after eggs were first observed (the same time frame as in the first nest). This allowed brood to develop for as long as possible, while ensuring no offspring eclosed, allowing us to control the number of workers in nests. All collected brood and adult bees were stored at −80°C until further processing.

Any queens that survived the duration of the experiment but did not lay eggs in the first (*n* = 2) or second (*n* = 1) nests were collected after 60 or 30 days, respectively, and were not included in statistical analyses. Queen mortality and the number of days until the first eggs were observed were recorded. Nests were dissected over dry ice and the number of eggs, larvae, and pupae were recorded.

#### Statistical analyses

GLMMs were used to determine predictors for two response variables: number of days until first eggs were observed in the nest and total number of brood items (eggs, larvae and/or pupae) in the nest. Details on model formation are provided in the Supporting Information. Best-fit models and *P*-values were identified according to the methods in Experiment 1. Mortality was analysed with a mixed-effects Cox regression model using the coxme package (v. 2.2–16; Therneau, 2014) and survival package (v. 3.1-12; Therneau, 2000), for which significance was calculated by performing an Analysis of Variance (anova()) on the best-fit model, and data were visualized using survminer (v. 0.4.0) and ggplot2 (v. 2.2.1).

## Results

### Experiment 1

#### Effect of workers on queen ovary development

**Figure 3:**
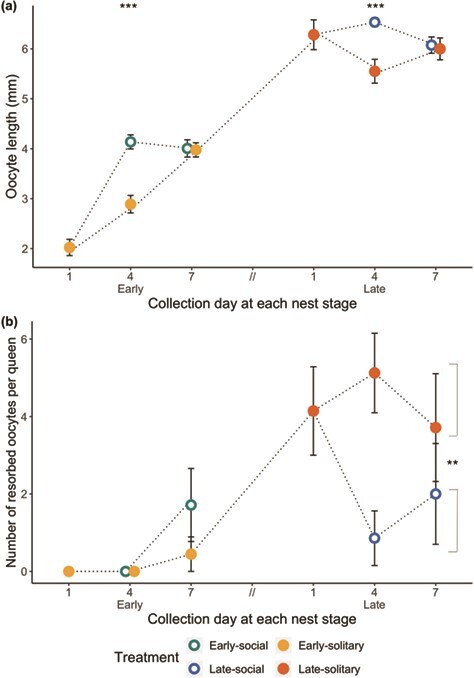
Ovary development in queens from Experiment 1. Dotted lines represent the social history of each treatment group. Overlapping points are horizontally jittered for easier visualization. (a) Mean oocyte lengths (+/− s.e.m.) for terminal oocytes. Asterisks represent *P*-values (^***^P < 0.001, *n* = 5–9 queens, 40–71 oocytes) for pairwise Welch’s two-sample *t*-tests between solitary and social queens at each timepoint. (b) Mean number of resorbed oocytes (+/− s.e.m) per queen. Late-social queens had significantly fewer resorbed oocytes than late-solitary queens (post hoc Tukey, ^**^P < 0.01, *n* = 12–24).

The presence of workers in the nest positively impacted queen ovary development, as evidenced by an increase in mean oocyte lengths (GLMM *P* = 0.027, estimate = 0.65, 95% CI [0.08, 1.22], Fig. 3a) and a decrease in variability among oocyte lengths in social relative to solitary queens (pairwise Levene’s tests: early Day 4, *P* < 0.001; early Day 7, *P* = 0.77; late Day 4, *P* < 0.001; late Day 7, *P* < 0.001). Maximum oocyte lengths did not differ between social and solitary queens at any single time point, but presence of workers was associated with a higher minimum (and therefore greater average) queen oocyte length on Day 4 in both early- and late-stage nests (pairwise Welch’s 2-sample *t*-tests: early Day 4, *P* < 0.001; late Day 4, *P* < 0.001; Fig. 3a). By Day 7 in both early- and late-stage nests, solitary and social queen oocyte lengths no longer differed (pairwise Welch’s two sample *t*-tests: early Day 7, *P* = 0.90; late Day 7, *P* = 0.79, Fig. 3a). The best-fit model predicting oocyte lengths included social treatment, nest stage, collection day, and the interaction between nest stage and collection day as fixed effects.

In the late-stage groups, the presence of workers was associated with decreased oocyte resorption, whereby late-social queens had fewer resorbed oocytes than late-solitary queens (GLMM pairwise, Tukey-adjusted lsmeans: *P* < 0.01, estimate = 4.65, 95% CI [3.00, 6.30], Fig. 3b). Because most early-stage queens did not yet have mature oocytes, oocyte resorption was infrequent in these groups and did not differ between early-social and -solitary queens (GLMM pairwise, Tukey-adjusted lsmeans: *P* = 0.096, Fig. 3b). The best-fit model predicting oocyte resorption included social treatment, nest stage and their interaction as fixed effects.

#### **Effect of workers on queen** JH **levels**

**Figure 4:**
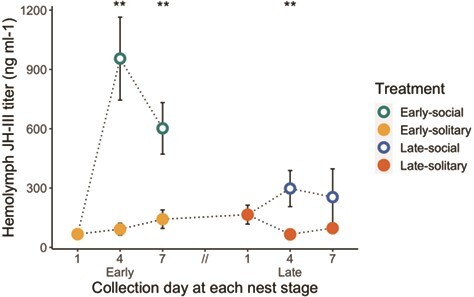
Mean JH titre (+/− s.e.m.) in queen hemolymph from Experiment 1. Dotted lines represent the social history of each treatment group. Asterisks represent Bonferroni-corrected *P*-values (^**^P < 0.01, *n* = 5–9) for pairwise Wilcoxon rank sum exact tests between solitary and social queens at each time point.

Social status strongly impacted queen JH titres, as the presence of workers in the nest resulted in elevated titres, irrespective of queen reproductive state (GLMM *P* < 0.001, estimate = 0.36, 95% CI [0.27, 0.45]; Fig. 4). There was a significant interaction between social status and nest stage, where JH levels in early-stage queens were more strongly impacted by social status than late-stage queens (GLMM *P* = 0.002, estimate = −0.21, 95% CI [−0.34, −0.08]; Fig. 4). Solitary queens maintained relatively low JH titres at all time points (all Bonferroni-adjusted pairwise Wilcoxon rank sum tests among solitary queens, *P* > 0.1), irrespective of reproductive state, although reproductive state, represented by oocyte length, was a weak predictor of JH titre independent of social status (GLMM *P* = 0.002, estimate = 0.05, 95% CI [0.02, 0.08]). The best-fit model predicting JH titre included social treatment, oocyte length, nest stage and the interaction between social treatment and nest stage as fixed effects.

#### Effect of workers on queen functional reproduction

**Figure 5:**
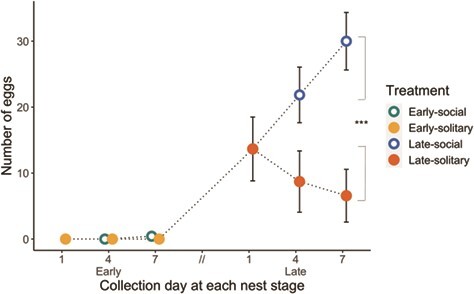
Mean number of eggs (+/− s.e.m.) in nests from Experiment 1. Dotted lines represent the social history of each treatment group. Overlapping points are horizontally jittered for easier visualization. No early-solitary nests contained eggs at any time point. Nests of late-social queens had significantly more eggs than those of late-solitary queens (post hoc Tukey, ^***^P < 0.001, *n* = 12–24).

Social nests contained significantly more eggs than solitary nests (GLMM *P* < 0.001, incidence rate ratio = 15388.64, 95% CI [52.85, 4480383.51]; Fig. 5). No eggs were detected in any of the 26 nests of early-solitary queens. Of the 7 early-social nests collected on Day 4, 1 contained eggs, and of the 7 early-social nests collected on Day 7, 3 nests contained eggs. Social and solitary early-stage nests did not differ from one another with respect to the number of eggs (GLMM pairwise, Tukey-adjusted lsmeans: *P* = 0.7, Fig. 5) and were excluded from additional statistical analyses because so few nests contained eggs. All late-social nests contained eggs, whereas eggs were detected in only 13 (62%) out of 21 of late-solitary nests (late Day 1, *n* = 5; late Day 4, *n* = 5; late Day 7, *n* = 3). Late-social queens had, on average, approximately twice as many eggs at Day 4 (mean +/− s.e.m. 21.86 +/− 4.21 eggs) and 4 times as many eggs at Day 7 (30.00 +/− 4.36) relative to late-solitary queens at matching time points (Day 4, 8.7 +/− 4.65; Day 7, 6.57 +/− 4.00). The best-fit model predicting egg number included social treatment, oocyte length, queen body size, collection day and the interaction between social treatment and oocyte length as fixed effects.

### Experiment 2

#### Effect of workers on queen functional reproduction

**Figure 6:**
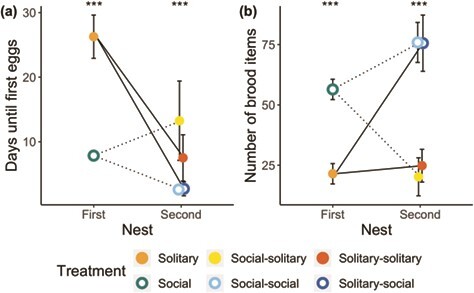
Functional reproduction in Experiment 2. Lines represent the repeated measures trajectory and social history of each treatment group: solid lines, first nest was solitary (solitary-solitary and solitary-social groups); dotted lines, first nest was social (social-solitary and social-social groups). Asterisks represent *P*-values (^***^P < 0.001, *n* = 14–19) for post hoc Tukey tests of all solitary versus all social nests at each time point. Overlapping points are horizontally jittered for easier visualization. (a) Mean number of days (+/− s.e.m.) until eggs were first observed. (b) Mean number of brood items (+/− s.e.m.) on the day of collection.

Social queens laid eggs sooner than solitary queens in their first and second nests, irrespective of social history (GLMM *P* < 0.001, estimate = 1.15, 95% CI [1.05, 1.25], Fig. 6a). Queens also laid eggs sooner in second nests relative to first nests, irrespective of social history (GLMM *P* < 0.001, estimate = −0.44, 95% CI [−0.80, −0.36]; Fig. 6a). Nest, social treatment and their interaction were included as fixed effects in the best-fit model to predict the number of days to lay eggs. The number of days until eggs were first observed was more variable in solitary nests relative to social nests (Levene’s test for equal variances *P* < 0.001).

Brood was observed in all social nests, but in only 85% of solitary first-nest and second-nest queens. Of the nests that did contain brood, social nests contained on average more brood items (eggs, larvae and/or pupae) than solitary nests (GLMM social treatment *P* < 0.001, estimate = 36.93, 95% CI [2.80, 33.48]; Fig. 6b). The number of brood items in the second nest was not impacted by social history (GLMM pairwise comparisons via Tukey-adjusted lsmeans social-solitary vs. solitary-solitary second nests, *P* = 0.94; social-social vs. solitary-social second nests, *P* = 0.99, Fig. 6b). The best-fit model predicting brood number included social treatment, social history, and their interaction as fixed effects.

#### Effect of workers on queen mortality

**Figure 7:**
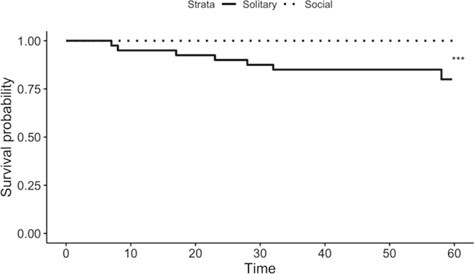
Queen mortality in Experiment 2. X-axis: number of days from the start of the nest. Y-axis: proportion of queens alive at the given time point. First and second nests are shown together on a single graph comparing solitary versus social queen survival. Only 1 queen from a second nest died (on Day 58, queen was 101 days old). Age at death for early nest queens = time + 13 (i.e. number of days in the first nest + age at the start of the first nest). Asterisks represent *P*-values (^***^P < 0.001, *n* = 36–40) for Cox regression model.

Solitary queens had higher mortality than social queens (mixed effects cox regression, *P* < 0.001, chisq = 49.9; Fig. 7), with the overwhelming majority of mortality occurring in the first nest (7 out of 8 total deaths; mixed effects cox regression, *P* < 0.001, chisq = 1149.25). No queens in the experiment died while in the presence of workers. Nest and social treatment were included as fixed effects in the best-fit model to predict mortality.

## Discussion

In social insects with a solitary nest-founding stage, the onset of the social stage is critical for nest survival, yet the mechanisms that facilitate this transition remain understudied. Here, we manipulated the social environment of early nesting queen bumble bees (*B. impatiens*) to explore how the life history transition from living solitarily to socially influences queen reproduction and survival. Our study is predicated on the hypothesis that the presence of workers accelerates queen functional reproduction in bumble bees (Röseler, 1968; Kwon *et al.*, 2006; Woodard *et al.*, 2013), thus aligning queen egg production with the emergence of helpers in the nest, who around this time assume the task of rearing offspring. This alignment is proposed to be adaptive, because it helps ensure nesting success by rapidly increasing the number of workers, and therefore the productivity (Malfi *et al.*, 2019), of the nest. Collectively, across both of our experiments, we found that queens in a social environment exhibit increased ovary activation, elevated JH titres, accelerated egg laying and higher survival, relative to solitary queens. This supports our hypothesis that workers positively impact queen fecundity and survival during the early nesting stage. However, we found that these positive effects are transitory, in that they reflect only the current social environment and not social history. Our experimental design also allowed us to uncouple the social environment from reproductive status, and our data demonstrate that queen JH levels are strongly positively impacted by the social environment irrespective of queen reproductive state.

### Bumble bee workers promote reproduction and survivorship in queens

In our study, queen ovarian development and egg laying were both increased by the artificial addition of workers to the nest. For example, eggs were observed, on average, 3-fold sooner in the first nests of social relative to solitary queens. Similarly, social queens laid on average 2–3 times as many eggs as their solitary counterparts, a finding also observed in the bumble bee *B. terrestris* (Woodard *et al.*, 2013). Conversely, we also observed reduced fecundity in queens who lacked workers in their nests at around the time point in the colony cycle that they would typically emerge. Specifically, we detected fewer eggs and more resorbed oocytes in relevant solitary nests (i.e. nests of late-solitary and social-solitary queens). These data are consistent with a previous study in *B. terrestris* that found that queens whose workers are removed upon emergence (much like the late-solitary group here) exhibit delayed reproduction and increased mortality relative to queens that retain their workers (Engels, 1990). Social insect queens have evolved to produce large numbers of eggs throughout their lives, with queens of some species laying hundreds of thousands, or even millions, of eggs over their lifetime (Winston, 1987). We propose that worker regulation of queen reproductive status is a related, under-studied aspect of eusocial evolution, particularly in lineages with solitary nest founding.

From an evolutionary perspective, queens may perceive workers in the nest as an honest indicator of helpers, and selection might have favoured the ability to adjust reproductive output accordingly. However, a queen’s first cohort of workers could be lost to events such as extreme weather, predation, or habitat destruction, and the ability to reverse worker-induced reproductive acceleration, as we observed in our experiments, may also be advantageous for queens. Thus, intensive selective pressures might have shaped the evolution of mechanisms that promote synchronization between the acceleration of queen reproduction and development of a social environment capable of rearing those offspring.

Given enough time (specifically, 7 days), solitary queens in our study ultimately did reach similar levels of ovary development as seen in social queens. However, that some social queens, and no solitary queens, were laying eggs at this time point suggests that in addition to their impacts on queen ovarian development, workers may also accelerate or release queen egg laying behaviour. Thus, reproductive maturation and egg laying behaviour may be controlled independently in the bumble bees, consistent with previous observations that workers sometimes develop their ovaries but do not lay eggs (Duchateau and Velthuis, 1989). Although we did not explicitly test this, the positive association between accelerated reproduction and increased survival in social queens in our study differs from what has been observed in many animals, in which trade-offs exist between longevity and fecundity (Stearns, 1992). This relationship is reversed in many eusocial lineages; reproductive queens often live orders of magnitude longer than non-reproductive nestmates (Carey, 2001). Indeed, one study in eusocial ants found that the activation of queen reproduction itself promotes longevity in queens, as reproductive queens outlived their non-reproductive counterparts, irrespective of their social environment (Rueppell *et al.*, 2015). From the results of our study, we are unable to determine whether queen reproductive activation (prompted by worker presence) promotes longevity, as it can in other eusocial insects (Rueppell *et al.*, 2015), or whether there is some alternative mechanism operating that enhances survival. However, the fact that we detected a difference in mortality in this buffered laboratory environment containing unlimited food and no exposure to predators or weather events suggests a physiological, rather than environmental, mechanism. Overall, our data are consistent with the hypothesis that in social insects, sociality promotes queen survival and resilience and decreases variation in the number of offspring queens produce, an idea thus far supported primarily by theoretical rather than empirical studies (Stevens *et al.*, 2007; Kennedy *et al.*, 2018). Our finding that social queens had the same maximum, but a higher minimum, oocyte length relative to solitary queens suggests that workers may advance queens towards a personal physiological maximum rate of reproduction. Additionally, our data suggest that, while queens can reinitiate new nests after losing or abandoning their offspring, they have no observed reproductive advantage over queens who are starting their first nests. Instead, the reproductive benefits of sociality are conditional on a continuous social input in this species. This finding highlights the importance of producing and maintaining early season workers, and therefore a reproductive and survival advantage, for queens in young nests.

### JH **is involved in bumble bee social organization**

JH likely mediates the accelerated reproduction observed in social queens in our study, which is consistent with its role as a gonadotropin in other insects (Roy *et al.*, 2018). However, JH titres were most strongly impacted by the social environment in our study, irrespective of queen reproductive state. In addition to its conserved role as an adult gonadotropin (Adams, 2009) and regulator of early-life development (Jindra *et al.*, 2013; Truman, 2019), JH has evolved to take on new functions in some insects. For example, it has been co-opted to play a role in reproductive dominance in many social insects (reviewed in Kapheim and Johnson 2017), although this has not yet been demonstrated in bumble bees. Previous studies on JH in bumble bees have focused almost exclusively on workers in the first week of their lives (Röseler, 1977; Röseler and Röseler, 1978; Duchateau and Velthuis, 1989; Bloch *et al.*, 2000a, 2000b; Bloch and Grozinger, 2011; Hartfelder *et al.*, 2013; Amsalem *et al.*, 2014; Shpigler *et al.*, 2014, 2016), or newly emerged gynes (young queens) prior to diapause (Röseler and Röseler, 1988; Amsalem *et al.*, 2014), rather than nesting queens (but see Amsalem *et al.* 2014). Further, in these previous studies, workers are always maintained in social groups (e.g. Bloch, Hefetz, *et al.* 2000a; Shpigler *et al.* 2014), and reproductive individuals in these groups are nearly always considered dominant. Here, by investigating JH in early nesting queens (rather than workers or gynes) across solitary and social conditions, we were able to uncouple the social environment from reproductive state to disentangle dominance, reproduction and social status.

JH may indirectly promote reproduction in social individuals through its involvement in dominance establishment (consistent with the ‘challenge hypothesis’; Tibbetts and Huang 2010). This is supported by our finding that *early-social* queens, which were not yet reproducing when workers were added to the nest and were likely in the process of establishing reproductive dominance (Amsalem and Hefetz, 2010), had higher JH titres than late-social queens, which were reproductively mature at the time of worker introduction and therefore may have been able to establish dominance more readily. Our data further suggest that relatively high JH levels are not necessary for oogenesis to proceed in solitary individuals. High JH levels are also not necessary for worker ovary development in social colonies (Röseler, 1977). Alternatively, the high JH levels observed in early-social relative to late-social queens in our study may indicate the involvement of JH in the initiation of egg laying. JH acts broadly on the insect nervous system (Fahrbach and Robinson, 1996; Anton *et al.*, 1999) and plays a major role in the control of oviposition behaviour and pheromone production in many insects (Nijhout and Wheeler, 1982). A more thorough investigation of the interaction between JH, oogenesis and oviposition across a broader spectrum of social configurations is needed to clarify this interaction.

A remaining question is how workers cause the observed changes in queen reproductive physiology, upstream of their effects on JH. In other eusocial insects, nestmates frequently communicate with, and socially regulate, one another through an array of chemical, visual, tactile and other signals (Billen, 2006). In bumble bees, brood attenuate queen circadian rhythmicity (Eban-Rothschild *et al.*, 2011), but beyond this, the social signals that impact queens are largely unknown. With respect to regulation of reproduction, tactile cues have been shown to stimulate reproduction in cockroach females (Uzsák *et al.*, 2014), whereas pheromones and aggressive interactions have been broadly shown to limit reproduction in eusocial workers (Van Oystaeyen *et al.*, 2014). Further, JH levels are regulated by factors such as temperature, nutrition and insulin signalling in other systems (Flatt *et al.*, 2005), any of which may be impacted by the social environment in bumble bees. Alternative to, or in addition to, these direct mechanisms, workers may elicit the observed physiological changes in queens indirectly, by altering the queen’s energy balance. As the workers take over brood care and nest maintenance tasks, this may free the queen to invest more energy into reproduction. In our study, queen reproductive status reflected the current social environment only, suggesting the social environment has immediate, but not persistent, effects on queen fecundity. Indeed, social-social queens reinitiated egg laying in their second nest almost immediately (range, 1–5 days) whereas social-solitary queens took an average of 13 days to do so (range, 3–46 days). Thus, the underlying mechanism promoting queen fecundity is seemingly reversible, and likely requires some continuous input.

Our results are based on one domesticated species of bumble bee, *B. impatiens*. Working with domesticated species is essential for the types of experiments we conducted, which are difficult to carry out in the field or with at-risk species. Although we cannot rule out the possibility that artificial selection in the domestication process has impacted our results, we think it unlikely that our results are an artifact of bumble bee domestication. Natural history notes observing accelerated queen reproduction in the presence of workers were first recorded in *B. terrestris* prior to domestication (Sladen 1912), suggesting that this phenomenon is present in some form in wild, undomesticated bees. Further, domestication might be predicted to dampen the observed effects, rather than enhance them. This is because, in the wild, queens experience additional stressors such as overwintering, foraging, nest defence and a shorter summer season in which to grow their colonies. Any of these added stressors may result in stronger selective pressures for workers to induce accelerated reproduction and increase survival of queens in the ways we have demonstrated. The relatively short (~100 years) process of domestication has minimized, or altogether eliminated, many of these natural stressors for captive, commercial bumble bee populations (Velthuis and van Doorn, 2006). For example, domesticated queens experience less selective pressure to expedite their reproduction in the early season, because they are not bound by the short summer season. This may in turn result in a dampening of worker-induced queen reproduction in domesticated relative to wild lineages.

## Conclusions

Bumble bee queens, like other annually social insects, initiate colonies in spring that will perish by fall, and there is a limited window of time for colonies to grow and ultimately produce reproductives (males and new queens). Multiple lines of evidence suggest that the earliest stages of colony development are especially important for ultimate colony growth and success. For example, early season resources have disproportionate impacts on colony growth and reproductive success (Williams *et al.*, 2012; Malfi *et al.*, 2019; Mola *et al.*, 2021). Colonies grow exponentially throughout the nesting season, and the number of workers produced directly corresponds to the number of reproductives produced (Crone and Williams, 2016). Thus, queens likely benefit from being able to rapidly establish nests in spring. This also is consistent with the pattern that bumble bee species that emerge from diapause and begin nesting earlier in the spring are less likely to be declining, relative to those that emerge later in the season (Williams *et al.*, 2009). This evidence, along with our finding that queen survival and reproduction increase upon emergence of the social environment, collectively suggest that intervention strategies that target this early nesting stage and promote the production and maintenance of early season workers are needed for effective conservation of this solitary nest-founding, social lineage.

Bumble bees are the most economically important native pollinators in North America (National Research Council 2007) and play essential roles in pollination networks in wild plant communities (Ollerton *et al.*, 2012; Brosi *et al.*, 2017). Early nesting queen bumble bees play a vital role in early season pollination of wild plants and crops such as blueberry, because they emerge early in the season when temperatures are relatively cool and few other pollinators are able to fly (Willmer *et al.*, 1994; Tuell and Isaacs, 2010). Despite the economic and ecological importance of early nesting queens, current conservation strategies focus primarily on supporting bumble bee colonies during the social phase of their life cycle (Goulson *et al.*, 2007). Thus, the needs and unique biology of early nesting queens remain largely unknown and unaddressed (but see Baron *et al*. 2017; Bogo *et al*. 2017; Kells and Goulson 2003; Leza *et al*. 2018; Tripodi and Strange 2019; Watrous *et al.* 2019; Costa *et al.* 2021), although this stage may represent a particularly important demographic stage for bumble bee populations. Solitary queens must both forage and perform all the tasks required for colony success and reproduction, so this stage may respond strongly to environmental stressors such as diminishing or degraded floral and habitat resources, urbanization, pesticide use, and higher temperatures. Ultimately, the sensitivity of this life stage may help explain global declines in bumble bee populations (Goulson *et al.*, 2007, 2015; Cameron *et al.*, 2011).

Given that workers regulate queen physiology in the ways we have demonstrated, the timing of worker emergence in the nest, as well as the maintenance of those workers, likely impacts queen fitness, colony developmental trajectories and ultimately nesting success in bumble bees. Thus, we propose that bumble bee conservation regimes should focus more heavily on the early nesting period to support the emergence and maintenance of early-season workers in young colonies. For example, ensuring ample, pesticide-free forage and nesting resources in the early spring, particularly in agricultural, urban and other degraded and disturbed habitats, is one concrete action that would be predicted to have substantial positive impacts on nesting success. Current conservation regimes often focus on mitigating stressors in mid-summer (Goulson *et al.*, 2007), but focusing on the early spring may be just as important, if not more important, for supporting bumble bee population success. Additionally, more research investigating the unique needs and stressors affecting early season queens is essential to developing targeted conservation regimes specific to this life stage. For example, the effects of increased environmental stochasticity (Lande 1993), potential phenological mismatches between queen emergence and floral blooms (Kudo and Cooper, 2019) and warming temperatures (Soroye *et al.* 2020), on early season queens remain open areas for future climate change-related research. A more in depth understanding of the impacts of parasites and pathogens on early season queens, specifically (as opposed to social colonies), is also needed (but see Mullins *et al.* 2020). Our findings highlight unique aspects of the solitary nest-founding stage in social insects and underscore the importance of conservation interventions that support this early nesting period.

## Funding

This work was supported by the National Institutes of Food and Agriculture [CA-R-ENT-5122-H to S.H.W.] and the National Science Foundation [1631776 to E.K.].

## Author contributions:

E.S. and S.H.W. conceived the ideas and experimental design. E.S., P.S., K.M. and S.H.W. designed and validated the methodologies. E.S., D.R. and P.S. collected the data. E.S. and P.S. analysed the data. E.S., N.Y. and S.H.W. interpreted the data. E.S. and S.H.W. led the writing of the manuscript. All authors contributed critically to the drafts and gave final approval for publication.

## Data availability:

Data and code will be available from the Dryad Digital Repository: https://doi.org/10.5061/dryad.jdfn2z383 upon acceptance for publication.

## Competing Interests:

The authors declare to have no competing interests.

## Supplementary Material

supplement_only_coab047
