# Supplementary material for: An organizing feature of bumble bee life history: worker emergence promotes queen reproduction and survival in young nests
Source: Conserv Physiol. 2021 Jun 29;9(1):coab047. doi: 10.1093/conphys/coab047 (PMC8242224; doi:10.1093/conphys/coab047)
Supplement: supplement_only_coab047 [file supplement_only_coab047.docx]

**SUPPORTING INFORMATION**

**Experiment 1**

*Bee rearing and experimental design*

Queens (n = 83) were sourced from seven out of the 13 total natal colonies with equal distribution of bees from each natal colony across social treatment groups. At this stage, queens were placed in small individual plastic containers (approximately W7 x D7 x H5 cm) until they were transferred to larger plastic nesting boxes (approximately W15 x D15 x H10 cm) within 24 hours of the second CO_2_ treatment. In both the *early-social* and *late-social* groups, workers (n = 130) were sourced from seven out of the 13 total natal colonies, with equal distribution of bees from each natal colony across social treatment groups. Workers added to a given nest box were sourced from a single natal colony that differed from that of the queen. Late-stage queens were between 48-97 days old at the time of first offspring eclosion and were randomly assigned to each treatment group. Late-stage queens were between 50-104 days old at the time of collection.

*Juvenile hormone quantification methods*

To determine the concentration of JH-III and its intermediates in samples, JH-III (Toronto Research Chemicals E589400, North York, ON, CA), its intermediates farnesol (Sigma-Aldrich F203) and methyl farnesoate (Echelon Biosciences S-0153, Salt Lake City, UT, USA), and the internal standard citronellol, were used to make four standard mixes (Table S1). The retention time for each compound was determined by running dilutions of pure standards in scan (Acquisition General) mode on a Thermo Scientific Trace 1310 gas chromatograph coupled with an AI 1310 autosampler and a TSQ Duo triple quadrupole mass spectrometer running in single quadrupole mode with data acquisition and processing controlled by Chromeleon 7 software. Following this, detection and quantification of standards using selected reaction monitoring (SRM) at the specified retention times was validated using the quantitation and confirmation ion transitions validated in Kai et al. 2018 (Table S2).

To determine retention times, 2 µl of sample was injected at 230°C (inlet temperature) in “splitless with surge” mode with constant helium carrier gas (purity = 99.999% UHP200) flow rate of 1.2 ml min^-1^, split flow rate of 25 ml min^-1^, and splitless time of 1 min. Purge flow rate was set to 50 ml min^-1^, and constant septum purge (surge pressure 20 psi and surge duration 1 minute) and vacuum compensation were selected. Gas saver mode was enabled with a gas saver flow rate of 25 ml min^-1^ and gas saver time of 2 minutes. Compounds were condensed onto, and eluted from, a Thermo Scientific TG-5MS columns (0.25 mm i.d. × 28.33 m, 0.25 µm film thickness). The column oven was initially held at 60°C for 1 minute, then increased to 160°C at a rate of 25°C min^-1^, followed by a 12°C min^-1^ ramp to 280°C. The run was stopped at 20 minutes. The autosampler syringe was washed for 3 cycles in acetone (Fisher Scientific A949-4) and hexanes before and after each injection. For triple quadrupole MS parameters, the temperatures of the transfer line and ion source were both held at 280°C. For determining retention times, the mass spectrometer was operated in electron ionization mode using general acquisition parameters (scan mode). Scanning within a mass range of 30-500 began at 3 minutes with dwell time set to 0.2.

Following identification of retention times, quantification of compounds in both standards and biological samples using SRM methods was performed according to ion transitions and operating parameters specified in Kai *et al.* 2018 and Table S2, with GC parameters as described above and the mass spectrometer operating as a triple quadrupole instrument. In between each standard or hemolymph sample we ran a blank injection of hexanes using a quick-ramp method with a higher maximum temperature to ensure sample contents were fully eluted from the column (splitless mode with inlet temperature 250°C, column oven initially held at 60°C for 1 minute, then increased to 300°C at a rate of 35°C min^-1^). JH intermediates (farnesol, methyl farnesoate) were detected as standards using the SRM method but were not subsequently detected in the hemolymph samples. A standard curve of JH-III was generated using the four standard mixes by correlating the spiked JH-III concentration (0, 10, 50, or 400 ng ml^-1^, Table S1) with the measured peak area ratio of JH-III / citronellol. JH-III concentration (ng ml^-1^) in each sample hexane extraction was calculated by scaling the peak area ratio of sample JH-III / citronellol to the standard curve. JH-III concentration (ng ml^-1^) in the original hemolymph was calculated as JH-III concentration (ng ml^-1^) in hexane extraction times 200 divided by hemolymph volume in µl (200 divided by hemolymph volume is the dilution factor in the hexane extraction process).

*Statistical analyses*

The log transformation of JH titers was analyzed with a gamma distribution (family = gamma, link = log) and included social treatment (solitary or social), oocyte length (averaged across the eight terminal oocytes for each bee), queen body size (average marginal wing cell length), nest stage (early or late), and collection day (1, 4, or 7) as possible predictors. Nest stage was included as a categorical variable (rather than using sampling date alone to model time), because individual queens did not all produce their first adult offspring at the same age or on the same timeline. Thus, the collection date is relative to offspring eclosion in the late-stage nests, and is not an absolute date. Oocyte length was analyzed with a gaussian distribution (family = gaussian, link = identity) and included social treatment, JH titer, queen body size, nest stage, and collection day as possible predictors. Number of eggs in the nest was analyzed with a binomial distribution (family = binomial) and included social treatment, average oocyte length, JH titer, queen body size, nest stage, and collection day as possible predictors. Queen natal colony was included as a random effect in all analyses, and individual queen was also included as a random effect in analyzing oocyte length, where there were eight measurements per individual (corresponding to the eight terminal oocytes).

**Experiment 2**

*Bee rearing and experimental design*

Callow queens used in this experiment were sourced from five out of the 14 natal colonies with equal distribution of each natal colony across treatment groups. Workers were sourced from 13 out of the 14 natal colonies with representation from at least three worker natal colonies (all different from that of the queen) in each experimental nest. Queens were between 39-90 days old at the start of their second nest and between 62-115 days old at the time of final collection. Any workers that died during the course of the experiment (n = 20 bees in 8 nests) were immediately replaced with callow workers from a source colony different from that of the queen.

*Statistical analyses*

The number of days until the first eggs were detected was log transformed and analyzed with a gaussian distribution (family = gaussian, link = “log”) and the statistical model included social treatment (social or solitary), social history (was previously social, was previously solitary, or NA), and nest (first or second) as possible predictors. Number of brood items was analyzed with a gaussian distribution (family = gaussian, link = “identity”) and included social treatment, social history, and nest as possible predictors. It is possible that the presence of workers impacted whether or not a queen produced brood at all, but had no impact on the number of brood in those nests that did produce brood. Thus, we analyzed the number of brood for only those nests that produced brood, to be more conservative (i.e., minimize our risk of Type I error) and to increase the specificity of our results. Queen natal colony and individual queen were included as random effects in all analyses. Best fit models were identified according to the methods in Experiment 1.

**
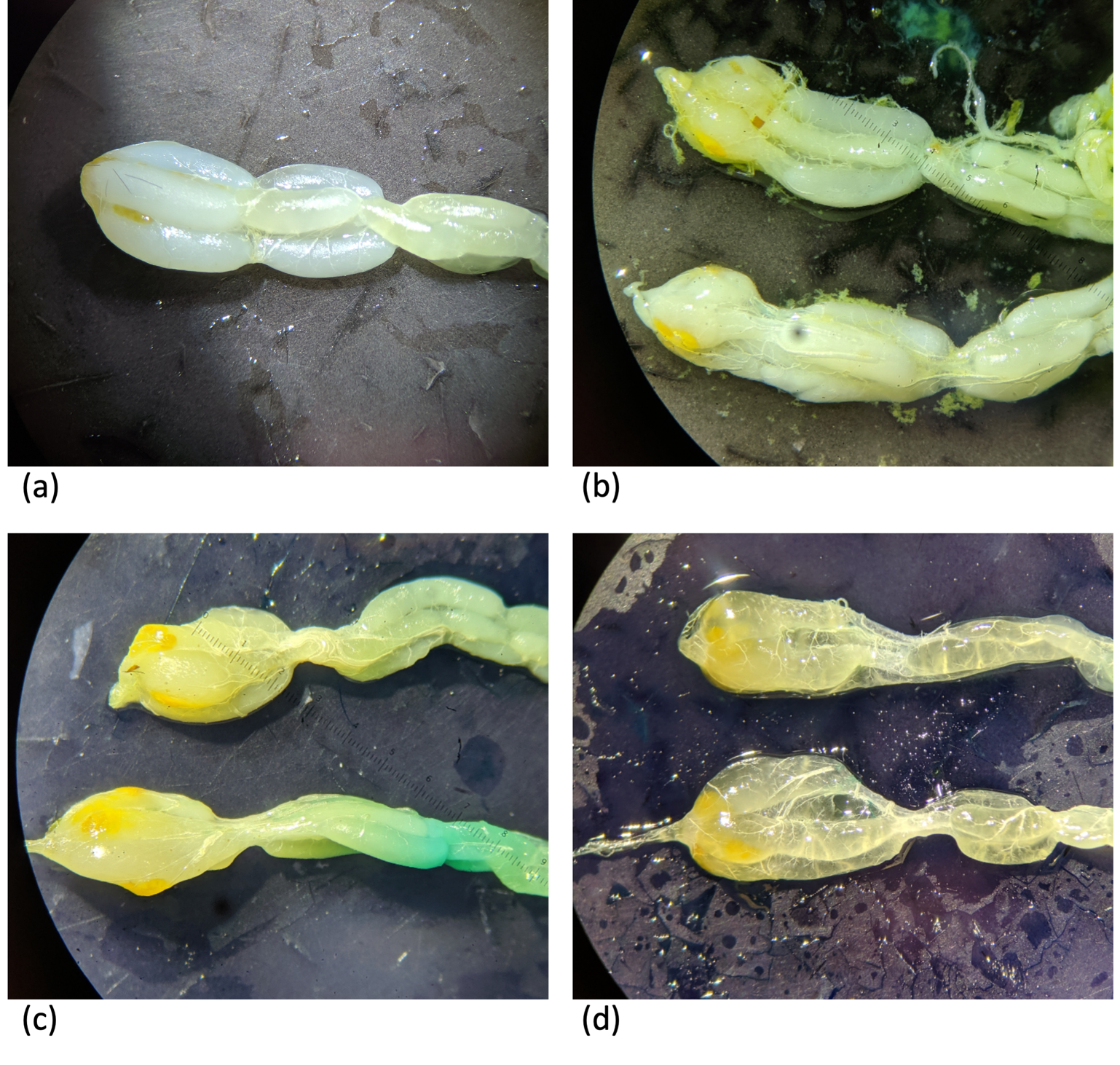
Figure S1. Resorbed versus unresorbed oocytes.** Mature, unresorbed oocytes (a) are uniformly shaped and white in color. Terminal ocytes in various states of resorption (b, c, d) can be identified by their yellow coloration and misshapen oocytes lacking a trophocyte.

**Table S1. Standard mix compositions used in JH-III quantification.**

| Standard Mix | Citronellol (ng ml^-1^) | JH-III  (ng ml^-1^) | Methyl farsenoate  (ng ml^-1^) | Farsenol  (ng ml^-1^) |
| --- | --- | --- | --- | --- |
| 1 | 100 | 0 | 0 | 0 |
| 2 | 100 | 10 | 10 | 20 |
| 3 | 100 | 50 | 50 | 100 |
| 4 | 100 | 400 | 400 | 800 |

**Table S2. MS Settings to quantify each compound in Acquisition-Timed mode on the GC-MS machine.**

| Compound | Retention Time | Ion Polarity | Window (min) | Pre-  width (min) | Post-Width (min) | Parent Mass | Product Mass | Collision Energy |
| --- | --- | --- | --- | --- | --- | --- | --- | --- |
| Citronellol | 5.37 | Positive | 0.1 | 0 | 0 | 81 | 79.1 | 10 |
| Farnesol Peak 1 | 8.77 | Positive | 0.2 | 0 | 0 | 93 | 77.1 | 15 |
| Farnesol Peak 2 | 8.96 | Positive | 0.1 | 0 | 0 | 93 | 77.1 | 15 |
| Methyl farnesoate | 9.45 | Positive | 0.1 | 0 | 0 | 114.1 | 83.1 | 10 |
| JH-III main peak | 10.33 | Positive | 0.1 | 0 | 0 | 85.1 | 59.1 | 10 |
